# Long-Term Consequences of Hepatitis C Viral Clearance on the CD4 (+) T Cell Lymphocyte Course in HIV/HCV Coinfected Patients

**DOI:** 10.1155/2015/687629

**Published:** 2015-01-26

**Authors:** J. Dazley, R. Sison, J. Slim

**Affiliations:** St. Michael's Medical Center, Seton Hall University, Newark, NJ 07102, USA

## Abstract

The long-term impact of pegylated-interferon plus ribavirin treatment outcome on CD4 T cell course in patients coinfected with human immunodeficiency virus and hepatitis C virus is largely unclear in the literature. The aim of this study was to investigate the impact of HCV-RNA clearance by standard anti-HCV therapy on long-term CD4 cells recovery in HIV/HCV patients on successful combined antiretroviral therapy. We retrospectively enrolled HIV/HCV-coinfected patients on HIV medications and treated for hepatitis C. CD4 + T cell counts were registered at baseline and after hepatitis C therapy. Multiple linear regression analysis was performed to identify independent predictors of CD4 + T cell change following the anti-HCV treatment outcome. Of the 116 patients enrolled, 54 (46.6%) reached a sustained virological response. During a follow-up of 24 months, the SVR group showed a mean annual increase in CD4 + T cell from baseline of 84 cells/ll at 1 year and of a further 38 cells/ll within the second year (*P* = 0.01, 0.001, resp.). An insignificant mean increase of 77 cells/ll occurred in the non-SVR group within month 24 (*P* = 0.06). Variables associated with greater CD4 gains were higher nadir, lower preinterferon CD4 counts, and lower body mass index (BMI).

## 1. Introduction

With the wave of rapidly evolving treatment of hepatitis C, it behooves us to understand its implications in our coinfected population, where the CD4 (+) T cell recovery after the introduction of an effective combined antiretroviral for HIV treatment is unclear in the literature. Studies have determined that HCV viremia did not affect the CD4 cell count recovery in the HIV patient [1].

Other previous studies have described coinfected patients that had results that implied a fall in the absolute CD4 count at week 24 of HCV treatment when interferon is used and later a subsequent rise to baseline at 24 weeks after the suspension of therapy [2–4]. What about longer periods of time after sustained virologic response? This is where there is a scarcity of data. The long-term effects of pegylated interferon plus ribavirin (Peg-IFN-RBV) treatment results on the CD4 T cell course in patients infected with HIV and hepatitis C virus are unclear in the current literature [5].

Our objective is to revisit the result of sustained viral response (SVR) by previously standard anti-HCV therapy on long-term CD4 cell count recovery in HIV/HCV patients on effective antiretroviral therapy (ART).

In short, we are asking the following question: what is the effect of long-term SVR on T cell counts in coinfected patients?

## 2. Materials and Methods

Sustained virological response (SVR) was defined by HCV-RNA PCR undetectability 6 months after IFN discontinuation. Now the standard of care defines SVR12 as an undetectable HCV RNA level 12 weeks after treatment discontinuation.

Non-SVR is defined by HCV active replication at the same time, independent of the reason (primary nonresponsive, relapse, and therapy discontinuation).

We retrospectively reviewed the charts of coinfected patients treated with pegylated interferon + ribavirin prior to January 1, 2008. Patients were divided into 2 groups based on their SVR24.

CD4 T cell modification is calculated as the mean change in CD4 (+) T cell counts from pre-IFN treatment values, which is the anti-HCV definition of response, and was evaluated after 6, 12, and 24 months (up to 60 months) status after IFN-based therapy discontinuation. The plasma HIV-RNA was recorded at the same time points to confirm viral suppression.

Subjects were enrolled into the study if (1) they had confirmed HIV and chronic hepatitis C and (2) HIV RNA VL was undetectable for at least 1 year on ART with continuous follow-up documented in our electronic medical records database.

Subjects included were treated between 2001 and 2008. CD4 (+) T cell counts were collected at baseline (before treatment), at end of treatment (EOT), then at every 6-month interval, and up to 60 months from therapy discontinuation, having a suppressed HIV virus load throughout the duration of hepatitis C infection therapy and beyond.

Multiple linear regression analysis was then implemented to identify independent predictors of CD4 (+) T cell change following the anti-HCV treatment outcome. Repeated linear regression was used to estimate the slope of the CD4 curve (i.e., change in CD4 count per year).

An independent samples *t*-test was used to compare continuous variables and Fisher's exact test was used to compare nominal variables. *P* value was considered significant at <0.05.

## 3. Results

Out of 205 coinfected patients treated, 26 SVR and 20 non-SVR patients were included in the analysis. Baseline characteristics were then constructed, as seen below. Reasons for excluding so many patients from the study were lost to follow-up after treatment, poor nonsuppressed and nonadherence to antiretroviral therapy, or HIV viral load detectability due to resistance.

Time to maximum CD4 count between the two groups did not differ (SVR = 2.88 years versus non-SVR = 2.95 years, *P* = 0.772). Thus, graphically, the subjects reached a plateau of their absolute CD4 counts and had no other increases or declines.

**Figure 1:**
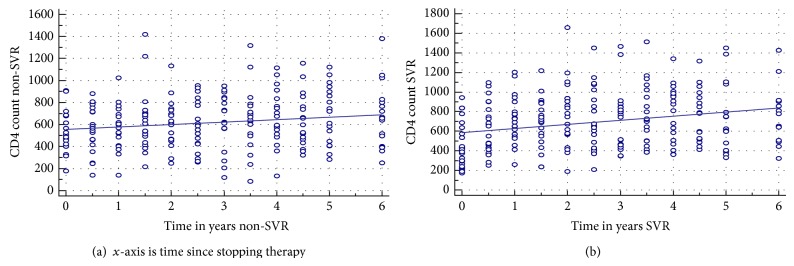
Scatter plots demonstrating the average of CD4 T cell counts for subjects over time in years for nonspontaneous virological responders (a) and spontaneous virological responders (b).

The slope (change in CD4 count per year) was significantly different between the two groups: SVR: increase of 83.8 in CD4 count per year (calculated to be the first 2.2 years) and non-SVR: increase of 44.9 in CD4 count per year (during first 2.2 years), *P* = 0.05. The long-term CD4 cell counts following the end of therapy showed an elevation in both groups, which was significant only in the SVR groups. There was no significant change in absolute CD4 count from baseline for the non-SVR group over 5 years, and the median value indicated that the levels of absolute CD4 counts were also similar. There were no differences in proportion of patients with significant fibrosis between the two groups.  Table 1 shows the basic characteristics for each subject and Table 2 indicates the CD4 count difference before and after HCV therapy.  Figure 1 demonstrates the natural slope seen at treatment start and continues for six years of SVR.

At baseline there were no subjects included in the study who had newly diagnosed diabetes mellitus II, lymphoma, or decompensated liver disease, and the same is true of follow-up. Upon follow-up during the study, there were 3 patients that died and 4 hospital admissions during the study dates. The deaths were due to cardiac causes, one from the SVR group and two from the non-SVR group. The hospitalizations were due to GI bleed, asthma exacerbation, chlamydia pneumonia, and MRSA bacteremia.

## 4. Discussion

A plausible explanation for the average of CD4 T cell counts having very little change over long periods of time may be the persistent immune activation and CD4 T cell apoptosis involved with HCV infection [6]. This activation decreases in response to IFN-RBV until 24 weeks after treatment interruption. This reduction in T cell activation may be the reason for the ongoing elevation in the CD4 count notwithstanding the success of the HCV treatment.

A lower nadir of CD4 cell count and higher preinterferon values may negatively affect the CD4 cell count increase after treatment has ended [7]. Due to splenic sequestration, cirrhosis and CD4 count variability has been polemic; however, one study used Fib-4 index, a correlation between the level of fibrosis and the CD4 count: [age (years) × aspartate(AST; IU/liter)]/platelet count (10^9^/liter) × ALT (IU/liter)^1/2^]^13^. It demonstrated a gradual increase with the fibrosis stage [8]. Increased fibrosis may be associated with a smaller rise in CD4 counts, which was not reproduced in our results.

The nadir of CD4 count prior to the start of treatment of HCV was not included, as it is possible that the nadir CD4 count drawn during treatment may be confounded by IFN induced leukopenia.

Greater BMI also gives a negative effect on CD4 cell count rise during the follow-up period [9]. Genotype 3 HCV may have impaired late immune recovery after ART initiation but is unclear [10]. Renal disease [11] and protease inhibitor monotherapy [12] also contribute in a negative way. Finally, there is also the burden of variability of the measurement of laboratory values performed at out of hospital facilities [13].

The key to understanding the relevance of our study and its applicability is to know the duration of HIV suppression [14]. The longer the duration, the more negligent the influence of HCV coinfection on the CD4 cell count [15], which may have been a factor in the flattening of the CD4 cell count gain in our study. There are studies showing that the higher the absolute CD4 count, the better the clinical outcome, but our study did not show such results by the descriptive data that was included.

The most relevant potential mechanisms that lead to a higher CD4 count when HIV subjects are treated for HCV and achieve SVR include the regression of liver fibrosis that may lead to the prevention of splenomegaly and cirrhosis. This may also be due to the effect of chronic inflammation in the non-SVR group leading to a depressed CD4 count (i.e., from HCV viremia).

## 5. Conclusions

The achievement of SVR may be associated with a significant immune reconstitution for 2 years after interferon and ribavirin treatment in HIV/HCV coinfected patients, with suppressed HIV viral loads. This phenomenon was not sustained past 2 years. Other factors mentioned should be analyzed to determine evidenced based ways to explain this finding and whether it benefits patients clinically.

Attaining SVR may be relevant with the implementation of direct acting agents (DAAs) now that we have very effective treatment and expect more patients to achieve a cure. This strengthens the rationale for treating patients with HCV, specifically coinfected HCV/HIV patients (i.e., achieving SVR may lead to better immunological condition for HCV-HIV patients).

As we continue to improve our screening capabilities for HCV, we are able to capture HCV patients earlier before much of the hepatic and extrahepatic damage and potentially not allowing the T cell nadir to be as low as it would have been now that the treatments are becoming simpler and shorter than ever.

The great majority of the 205 subjects were not included in the analysis for three reasons: they did not have controlled viral loads, they had unacceptable deficits in their data for collection, and there were a number of subjects that were lost to follow-up. The spreadsheets above indicate that all subjects in both groups were confirmed to be taking ART and had an undetectable virus load.

Our study is limited by the retrospective manner in which it was designed. Although it added liver histologies that previous studies did not consistently add and we had greater numbers than many of similar quality, more numbers would still be better. As such, even though we included a greater quantity of time after end of treatment was reached than that in many of the previous studies, a longer duration would even be better.

## Figures and Tables

**Table 1:** Basic characteristics.

Variable	SVR (*n* = 26)	Non-SVR (*n* = 20)	*P* value
Age, years	49.5 (IQR 46–55)	50.5 (IQR 47–55)	0.62
Male sex	21 (84%)	11 (55%)	0.19
Nadir CD4+, cells/ll	266 (IQR 206.5–377.5)	256 (IQR 200–413.5)	0.84
Baseline, T cells/ll CD4+ absolute count cell/mm^3^	501 (IQR 406–617)	620.5 (IQR 446.5–701.5)	0.17
HCV genotype 1	20 (77%)	17 (85%)	0.71
Significant fibrosis (F3-F4) (Scheuer)	13 (50%)	14 (70%)	0.33
African American race	15 (56.5%)	10 (50%)	0.76
Baseline HCV viral load IU/mL	669,000 (IQR 195,707–1,816,655)	500,000 (IQR 155,637.5–1,095,135)	0.66

**Table 2:** 

Time	SVR group mean CD4	Non-SVR group mean CD4
Baseline (prior to treatment)	358.75	619
At EOT	403.76	473.6
One year after EOT	724.17	582.9
Three years after EOT	706.62	661.9
